# DOCK 8 Deficiency, EBV+ Lymphomatoid Granulomatosis, and Intrafamilial Variation in Presentation

**DOI:** 10.3389/fped.2017.00038

**Published:** 2017-02-28

**Authors:** Victoria R. Dimitriades, Vincent Devlin, Stefania Pittaluga, Helen C. Su, Steven M. Holland, Wyndham Wilson, Kieron Dunleavy, Nirali N. Shah, Alexandra F. Freeman

**Affiliations:** ^1^Department of Pediatrics, Division of Infectious Diseases, Immunology & Allergy, University of California Davis Medical Center, Sacramento, CA, USA; ^2^Department of Pediatrics, Louisiana State University Health Sciences Center, New Orleans, LA, USA; ^3^Department of Pathology, NCI, NIH, Bethesda, MD, USA; ^4^NIAID, NIH, Bethesda, MD, USA; ^5^NCI, NIH, Bethesda, MD, USA

**Keywords:** DOCK8, lymphomatoid granulomatosis, lymphomatous granulomatosis, eBV lymphoproliferation, bone marrow transplantation

## Abstract

Dedicator of cytokinesis 8 (DOCK8) deficiency is an autosomal recessive, combined immunodeficiency within the spectrum of hyper-IgE syndromes. Epstein–Barr virus-positive lymphomatoid granulomatosis (LYG) (EBV + LYG) is a rare diagnosis and a previously unreported presentation of DOCK8 deficiency. A 10-year-old girl was initially evaluated for mild eczema and recurrent sinopulmonary infections. She had normal immunoglobulins with elevated IgE, poor polysaccharide response with low switched memory B cells, low CD4 count, and normal mitogen and antigen responses. Despite clinical improvement following immunoglobulin replacement, a prolonged cough prompted a CT scan, which showed nodules. Biopsy identified a Grade 2 EBV + LYG. Due to an inadequate response with chemotherapy, further workup for primary immunodeficiency was performed. With her symptoms of eczema and IgE elevation, along with her brother’s history of recurrent sinopulmonary infections and warts, targeted sequencing of DOCK8 was performed revealing compound heterozygous mutations for the two siblings. Both patients were successfully transplanted with resolution of the LYG and warts, respectively. This is the first reported case of LYG in DOCK8 deficiency. The EBV-driven lymphoproliferative disease along with the infection history in the brother led to the diagnosis of DOCK8 deficiency and curative hematopoietic stem cell transplants.

## Background

Dedicator of cytokinesis 8 (DOCK8) deficiency is a combined immunodeficiency, which was initially described as an autosomal recessive hyper IgE immunodeficiency syndrome (AR-HIES) (1). Unlike the more common autosomal dominant hyper-IgE syndromes (HIES) caused by loss of function mutations in *STAT3*, DOCK8 deficiency lacks the osseous and connective tissue defects that are prominent within the described phenotype of AD-HIES. Instead, patients with DOCK8 deficiency have increased risk of specific viral, bacterial, and fungal infections as well as prominent allergies and malignancies (1–3). Typically, DOCK8 deficiency is associated with significant cutaneous manifestations including disseminated HPV, molluscum contagiosum, recurrent herpes and varicella zoster. Malignancy is frequent in DOCK8 deficiency, predominantly, lymphoma and squamous cell carcinomas, and often leads to early mortality. In a large series of patients, mortality was over 50% by age 20 years (2).

Due to the high rates of morbidity and mortality, hematopoietic stem cell transplant (HSCT) is the treatment of choice and leads to resolution of many of the disease complications (4, 5). However, due to the multiple and variable initial presentations, DOCK8 deficiency may go undiagnosed for many years, leading to increased end organ damage. We present two siblings with DOCK8 deficiency identified initially through presentation of EBV+ lymphomatoid granulomatosis (LYG) in the lung in the younger sibling, a previously unreported association.

## Case Report

### Case #1

A 10-year-old girl first presented with an isolated third cranial nerve palsy associated with pansinusitis. She had a history of allergic rhinitis, mild atopic dermatitis, and a remote history of recurrent otitis media. There were no associated skin infections, bone abnormalities, or tooth retention. Laboratory examination revealed an IgE of 4,430 IU/mL with sensitizations to dust mites and *Candida*. Over the next few years, the patient developed recurrent sinopulmonary infections leading to further immunologic evaluation including normal IgG, normal IgA, slightly low IgM, and elevated IgE levels. Pneumococcal antibodies were protective in only 1/14 serotypes with low switched memory B cells; she responded poorly to both pure bacterial as well as conjugated polysaccharide vaccinations. Lymphocyte subpopulations showed low CD4 levels (absolute number 410), but mitogen and antigen testing were normal. She was diagnosed with a combined immune deficiency and started on monthly immune globulin replacement and trimethoprim/sulfamethoxazole prophylaxis. CT scan of the chest showed mild fusiform bronchiectasis in her lower left lobe. Sequencing of STAT3 for AD-HIES was wild type. For the next several years, she remained stable with few breakthrough infections.

**Figure 1:**
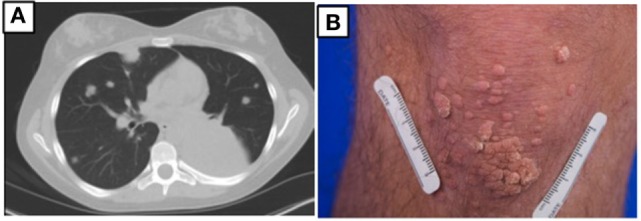
**(A)** Chest CT prior to hematopoietic stem cell transplant for patient 1 showing multiple masses due to EBV lymphoproliferative disease. **(B)** Multiple warts on the knee for patient 2.

**Figure 2:**
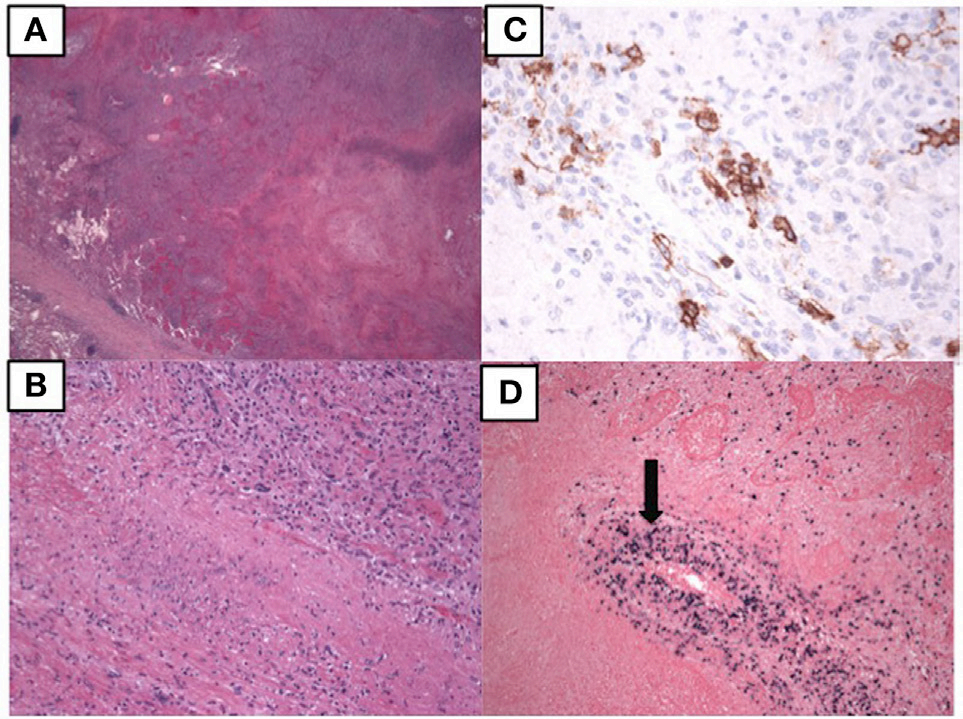
**Pathology of the lung biopsy of the right lower lobe consistent with lymphomatoid granulomatosis**. **(A)** 2× image showing large areas of necrosis. **(B)** 20× image showing lymphohistiocytic infiltrate with some large atypical lymphoid cells. **(C)** 40× image showing CD20 positive B lymphocytes by immunostaining. **(D)** 10× EBER showing EBV positive cells, including those in the vessel wall (arrow).

At age 14, a persistent cough led to repeat CT scan, which showed two new areas of nodularity (1–2 cm) in her left lower lobe (Figure 1). Pulmonary function testing was normal. Bronchoscopy was unremarkable, but due to the nodular appearance, she was started on empiric antifungal therapy with voriconazole. Rapid enlargement of bilateral lesions led to wedge biopsy, showing EBV+ Grade 2 LYG (Figure 2). Given the rarity of EBV + LYG and her age, she was referred to the National Cancer Institute for evaluation and treatment. Interferon alpha response to the LYG was minimal. Combination chemotherapy with etoposide, prednisone, vincristine, cyclophosphamide, doxorubicin, and rituximab (EPOCH-R) also elicited minimal response, and the LYG was thought to have evolved into a treatment-refractory lymphoma. In an attempt to explain poorly controlled EBV, eczema, and recurrent sinopulmonary infections with B cell failure, targeted genetic testing was done, which identified compound heterozygous mutations in *DOCK8*, c.1805G>A, p.W602X and c.4540delG, p.E1514KfsX8. Of note, she had mild atopic dermatitis, a small number of warts (flat and verrucous) and no history of molluscum. The patient and family gave written consent in accordance with the Declaration of Helsinki for an IRB approved clinical protocol for patients to receive bone marrow transplants (NCT01176006). She received a matched allogeneic stem cell transplant supplemented with donor-derived EBV-specific cytotoxic T lymphocytes, with complete regression of her EBV-related malignancy [Patient#5 in Cuellar-Rodriguez et al. (5)]. Three years after transplant, she has minimal sinopulmonary infections and normal IgG, IgM, and IgE with slightly low IgA levels. She sustains a robust response to repeat pneumococcal vaccination and her lymphocyte subpopulations have completely normalized.

### Case #2

Case 1’s older brother had a history of recurrent pneumonias and at age 5 years developed an empyema requiring a chest tube. Evaluation at that time revealed normal IgG, IgA, and IgM but an elevated IgE at 2,834 IU/mL. Both pneumococcal and *H. influenzae* B titers were low but he responded well to booster vaccinations. He was treated symptomatically for atopic disease (including conjunctivitis, rhinitis, and atopic dermatitis). His atopy continued and he was treated for multiple skin and soft tissue infectious in the setting of his atopic dermatitis, which was mildly responsive to corticosteroid treatment. Examination at age of 17 years found widespread warts on his elbows, knees, and the nape of his neck (Figure 1) and, during evaluation, he reported ongoing onychomycosis. Immunologic evaluation at that time showed normal IgG, IgA, and IgM with an elevated IgE of 4,368 IU/mL. There was low protection in response to both pneumococcal and tetanus vaccines, low switched memory B cells, and a low CD4+ T cell count (absolute number 385/μL) (Table 1). He had intermittent low grade EBV viremia and his pulmonary imaging was only remarkable for reactive lymph nodes in the axilla and retroperitoneal areas. In view of his sister’s diagnosis, at age 20 years, he was tested and found to have the same compound heterozygous DOCK8 mutations. After diagnosis, he was placed on antibiotic prophylaxis with trimethoprim/sulfamethoxazole until he successfully received a haploidentical HSCT on the same clinical trial of transplantation for patients with DOCK8 deficiency. He had a fairly unremarkable transplant course, and at 18 months posttransplant, the majority of his warts have resolved. Both parents were found to be heterozygous asymptomatic *DOCK8* mutation carriers.

**Table 1:** **Immunologic laboratories for both patients prior to HSCT**.

	Patient 1, 15 years (prior to lymphomatoid granulomatosis therapy)	Patient 2, 18 years	Normal
CD3 cells/μL	997	1,734	615–2,348
CD4 cells/μL	220	356	334–1,565
CD4/CD62L/CD45RA		57	102–1,041
CD4/CD62L/CD45RA−		109	162–614
CD4/CD62L−/CD45RA+		0	0–29
CD4/CD62L−/CD45RA−		188	42–225
CD8 cells/μL	471	1,264	149–853
CD8/CD62L/CD45RA		43	85–568
CD8/CD62L/CD45RA−		100	25–180
CD8/CD62L−/CD45RA+		227	11–172
CD8/CD62L−/CD45RA−		894	24–175
CD19 cell/μL	416	270	59–329
CD19/CD27	0.4%	0.6%	5–18%
NK cells/μL	405	268	109–607
IgG mg/dL	1,240^a^	2,070	716–1,711
IgA mg/dL	103	176	47–249
IgM mg/dL	<21	67	15–188
IgE (IU/mL)	1,890^b^	4,223	0–90

*^a^On IgG replacement therapy*.

*^b^IgE was evaluated at 10 years*.

## Discussion

Mutations in DOCK8 were described as the cause of many cases of autosomal recessive hyper IgE syndrome in 2009 (1). *DOCK8* is one of the DOCK180 family of exchange factors, which are responsible for activating Rho-family GTPases such as RAC and CDC42, and play crucial roles in cell division, survival, adhesion, migration, activation and differentiation (6). DOCK8 protein is expressed primarily in lymphocytes; its absence results in a combined immunodeficiency, characterized by impaired lymphocyte survival, migration and synapse formation (7–9). The increase in skin viral infections appears to be due to cytoskeletal defects that impair migration through the skin matrix (10), while the elevated rate of EBV infection and lymphoma likely relates to the poor NK function.

DOCK8 deficiency differs from dominant negative *STAT3* mutation because of the lack of connective tissue and skeletal abnormalities, with an increase in atopy and cutaneous viral infections. Although there is an increase in malignancy in STAT3 deficiency, the incidence of malignancy and early mortality in DOCK8 deficiency is much higher, with early onset of lymphomas (both EBV+ and EBV−) and HPV+ squamous cell carcinomas (1–3). Case 1 had early EBV+ lymphoproliferative disease presenting as LYG, a novel manifestation. She further differed from most DOCK8-deficient patients by having only minimal atopic dermatitis and viral skin infections. Her brother’s history was more typical, with increased cutaneous viral infections and atopic dermatitis, despite minimal sinopulmonary infections in later childhood.

Although EBV+ and EBV− lymphomas have been reported in DOCK8 deficiency, this is the first report of LYG, a rare EBV+ lymphoproliferative disease, which almost always affects the lungs. Like in our patient, it typically presents with pulmonary nodules (11). Disease is most common between the fourth and sixth decades with a male predominance, but earlier presentations are being increasingly recognized. Some level of immune dysregulation is thought to predispose to the poor EBV control leading to LYG, and some patients have had prior autoimmunity or immunodeficiency. Diagnosis is often delayed due to the predominantly pulmonary presentation, which is somewhat unusual for a lymphoproliferative process. Treatment is often aimed at augmenting the immune response, as was done in our patient with IFN alpha therapy, but there can be progression to lymphoma, as was noted in our patient. Although immunodeficiency is infrequently identified in patients with LYG, the early age of onset and poor response to therapy in Case 1 argued in its favor.

These siblings illustrate key features of DOCK8 deficiency, including the phenotypic variation regarding atopy, sinopulmonary infections, and cutaneous viral infections. In part, this may relate to reversion mutations with possible somatic mosaicism within certain cell lineages; however, this likely is not the only explanation. Although the reversion mutations may improve the disease progression, ultimate outcomes are similar to patients without reversions, so HSCT still should be the treatment of choice (12). Indeed, despite the reversion mutations found in both of our patients [patients #14 and 15 in Jing et al. (12), with #15 being the patient with LYG], the EBV lympholiferative disease still progressed, necessitating transplant for ultimate cure. These cases also highlight a novel manifestation of malignancy in DOCK8 deficiency and the benefit to early diagnosis and bone marrow transplant. Our index patient failed the typical therapy for EBV + LYG, leading to the consideration of a primary immunodeficiency and a personalized transplant plan. Despite the conventional approach to allogeneic HSCT in lymphoma, where it is ideal for patients to be chemoresponsive or in remission prior to transplant, she had active disease during the transplant conditioning regimen and her tumors regressed only after HSCT.

Although children with DOCK8 deficiency may do well for years, the disease is associated with significant morbidity and early mortality, regardless of the severity of skin involvement or immunoglobulin derangement. Curative HSCT should be considered early in the course of DOCK8 deficiency, and active EBV lymphoproliferative disease should not prevent patients from being considered for transplant.

## Ethics Statement

This study was carried out in accordance with the recommendations of NIAID and NCI IRBs at the NIH with written informed consent from all subjects. All subjects gave written informed consent in accordance with the Declaration of Helsinki. The protocols were approved by the NIAID and NCI IRBs.

## Author Contributions

VRD, VD, AF wrote the primary manuscript, were involved in the management of the patients, and conceived of the case report. SP was the pathologist who took the pictures and wrote the descriptions of the pathology figure. HS and her lab did the sequencing of the relevant gene to determine the diagnosis of the patients, and edited the paper. WW, KD, SH, and NS were involved in the clinical management and edited the manuscript.

## Conflict of Interest Statement

The authors declare that the research was conducted in the absence of any commercial or financial relationships that could be construed as a potential conflict of interest.
